# Arginine cluster introduction on framework region in anti‐lysozyme antibody improved association rate constant by changing conformational diversity of CDR loops

**DOI:** 10.1002/pro.4745

**Published:** 2023-09-01

**Authors:** Shingo Maeta, Makoto Nakakido, Hiroaki Matsuura, Naoki Sakai, Kunio Hirata, Daisuke Kuroda, Atsushi Fukunaga, Kouhei Tsumoto

**Affiliations:** ^1^ Bio‐Diagnostic Reagent Technology Center Sysmex Corporation Kobe Japan; ^2^ Department of Bioengineering, School of Engineering The University of Tokyo Tokyo Japan; ^3^ Department of Chemistry and Biotechnology, Graduate School of Engineering The University of Tokyo Tokyo Japan; ^4^ Life Science Research Infrastructure Group, RIKEN SPring‐8 Center Saitama Japan; ^5^ Research Center for Drug and Vaccine Development National Institute of Infectious Diseases Tokyo Japan; ^6^ Institute of Medical Science The University of Tokyo Tokyo Japan

**Keywords:** encounter complex, fab, molecular dynamics simulation, SPR, transition state, X‐ray crystallography

## Abstract

Antibodies are used for many therapeutic and biotechnological purposes. Because the affinity of an antibody to the antigen is critical for clinical efficacy of pharmaceuticals, many affinity maturation strategies have been developed. Although we previously reported an affinity maturation strategy in which the association rate of the antibody toward its antigen is improved by introducing a cluster of arginine residues into the framework region of the antibody, the detailed molecular mechanism responsible for this improvement has been unknown. In this study, we introduced five arginine residues into an anti‐hen egg white lysozyme antibody (HyHEL10) Fab fragment to create the R5‐mutant and comprehensively characterized the interaction between antibody and antigen using thermodynamic analysis, X‐ray crystallography, and molecular dynamics (MD) simulations. Our results indicate that introduction of charged residues strongly enhanced the association rate, as previously reported, and the antibody–antigen complex structure was almost the same for the R5‐mutant and wild‐type Fabs. The MD simulations indicate that the mutation increased conformational diversity in complementarity‐determining region loops and thereby enhanced the association rate. These observations provide the molecular basis of affinity maturation by R5 mutation.

## Abstract 2

PDB Code(s): 8GQ1;

## 1 INTRODUCTION

Antibodies are used for many therapeutic and biotechnological purposes, such as cancer immunotherapy, companion diagnosis, and molecular biosensors (Adair, 1992; Lawson, 2012; Muda et al., 2011). Their use is greatly facilitated by good biophysical properties, including high specificity and affinity toward antigens. Antibodies acquire such binding properties by altering the amino acid composition in the six hyper‐variable regions known as complementarity‐determining regions (CDRs) (Kabat et al., 1976). CDRs consist of a small number of amino acids and encode unique structural diversity and provide antibodies with the ability to recognize a wide variety of target antigens (Kabat et al., 1977).

The affinity of an antibody to the antigen is critical for clinical efficacy of pharmaceuticals and sensitivity of diagnostic reagents (Adams et al., 1998; Kempeni, 1999; Roovers et al., 1998; Kirsch et al., 2008). To obtain antibodies with high affinity, many affinity maturation methods have been developed, including phage display and rational molecular design (Yang et al., 1995; Tang et al., 2021). Another affinity maturation strategy is fluctuation editing, which enhances the affinity of antibodies by modulating the molecular fluctuation by amino acid mutations (Yanaka et al., 2017). However, it is essential to identify structural data and/or hot‐spots in order to apply these affinity maturation strategies (Akiba et al., 2019).

Previously, we reported an affinity maturation strategy in which the association rate of the antibody toward its antigen is improved by introducing charged amino acid residues into the framework region of the antibody (Fukunaga and Tsumoto, 2013). In contrast to conventional strategies, this approach does not involve precise characterization of the interaction between the targeting antibody and antigen. Therefore, this approach can be applied to a variety of antibodies. Recently, we reported that an engineered antibody developed using this technology exhibited favorable enthalpic changes in the transition state, implying that the improved interaction was not brought about by simple electrostatic attraction (Fukunaga et al., 2018).

Previous studies have indicated that affinity maturation in the immune system is associated with the loss of flexibility in the CDRs (Fernández‐Quintero et al., 2020; Thorpe and Brooks, 2007). Also, it has been suggested that the understanding of dynamic protein structure would be essential to design antibody (Löhr et al., 2022; Fernández‐Quintero et al., 2021). Therefore, consideration of dynamic structural changes is important for understanding the mechanism of affinity improvement (Jeliazkov et al., 2018). In this study, we applied our strategy to a Fab fragment targeting hen egg white lysozyme, which possesses a highly positive surface charge, and comprehensively characterized the antibody–antigen interaction using thermodynamic analysis, X‐ray crystallography, and molecular dynamics (MD) simulations to assess the molecular mechanism responsible for the improved affinity. Our results suggest that introduction of the arginine cluster into the framework region did not alter the overall complex structure but did affect the fluctuation of the CDR loops and increased the conformational diversity, thereby enhancing the association rate. Therefore, our strategy is a promising approach to improving antibody affinity.

## 2 RESULTS

### 2.1 Kinetic and thermodynamic parameters of the arginine mutant

**FIGURE 1:**
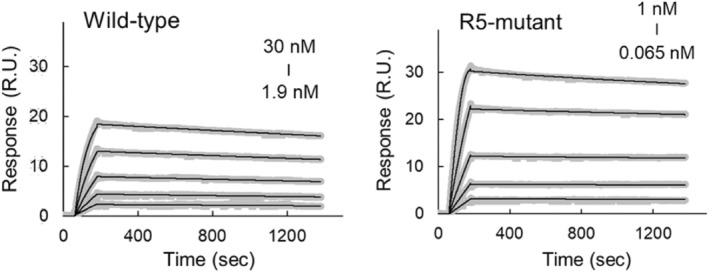
SPR sensorgrams of Fab and antigen binding. Sample concentrations are lower for R5‐mutant (1.9–30 nM for wild‐type and 0.065–1 nM for R5‐mutant). Wild‐type and R5‐mutant were injected over the surfaces on immobilized HEL protein. Running buffer injection was subtracted as the blank (gray: raw data; black: fitting data). Sample concentrations are lower for R5‐mutant (1.9–30 nM for wild‐type and 0.065–1 nM for R5‐mutant).

To evaluate the effect of the arginine mutation on binding affinity, we conducted surface plasmon resonance (SPR) analysis and determined kinetic parameters of the interaction between the Fab and antigen. Figure 1 shows the SPR sensorgrams, and the kinetic parameters for the wildtype and R5‐mutant are summarized in Table 1. The *k*_on_ value of the R5‐mutant was 68.6 times greater than that of the wild‐type, whereas the *k*_off_ value was almost the same as that of the wild‐type, indicating that the arginine mutation mainly influenced association.

**TABLE 1:** Kinetic parameters for wild‐type and R5‐mutant.

Analyte	*k*_on_ (M^−1^ s^−1^)	*k*_off_ (s^−1^)	*K*_d_ (M)
Wild‐type	(4.9 ± 0.01) × 10^5^	(1.1 ± 0.01) × 10^−4^	2.3 × 10^−10^
R5‐mutant	(3.3 ± 0.01) × 10^7^	(9.4 ± 0.02) × 10^−5^	2.8 × 10^−12^

HyHEL10 has multiple tyrosine residues which are involved in the antigen recognition as hotspot residues, Tyr33 and Tyr50. Both hotspots are found to be critical because the mutants, in which the residues were mutated to Ala, completely lost binding activity (Shiroishi et al., 2007). In more detail, the mutation of Tyr 33 to Phe maintained the binding, indicating that the aromatic ring is a key component. On the other hand, the mutation of Tyr 50 to Phe significantly reduced binding activity, suggesting the major contribution of hydroxyl group of Tyr. To assess if the CDR based specificity is maintained in the presence of R5 mutation, we evaluated the effect of R5 mutation on those hot spot mutants. We first prepared hotspot mutants by mutating each Tyr to Phe/Ala, namely HY33F, HY33A, HY50F and HY50A, respectively. Then we introduced R5 mutations to the hotspot mutant, denoted as R5‐HY33F, R5‐HY33A, R5‐HY50F and R5‐HY50A. The results of SPR sensorgrams are shown in Figure S1. The HY33F mutant maintained binding activity although the affinity was decreased, which is consistent with the previous study (Shiroishi et al., 2007). On the other hand, the binding response was significantly decreased for HY50F, which is also consistent with the previous study. Subsequently, we analyzed the Arg mutants and R5‐HY33F showed higher affinity with the improvement of *k*_on_. Regarding R5‐HY50F, the response was increased compared to HY50F although the parameters were not able to be determined, suggesting that the R5 mutation would improve the binding activity as with the other mutants. As for the Ala mutations, the binding of the antibody was completely lost in both HY33A and HY50A (Figure S1c). Although small binding response was observed for the mutants with R5 mutation, the binding affinity was not recovered. These results indicate that the hotspot residues are essential for antigen binding even in the presence of R5 mutation and the R5 mutation would not alter antigen binding specificity.

Subsequently, we conducted SPR analysis with increasing temperatures and calculated enthalpic changes upon interaction. In the transition state (Δ*H*‡) and the bound state (Δ*H*), we calculated enthalpy of the interaction using Eyring's plot and van't Hoff's plot, respectively (Table 2). At the transition state, the wild‐type showed unfavorable enthalpic changes (Δ*H*‡ > 0), whereas the R5‐mutant showed favorable enthalpic changes (Δ*H*‡ < 0). These results indicate that the R5‐mutant bound to the antigen through different processes compared to the wild‐type, resulting in the faster association.

**TABLE 2:** Comparison of enthalpy changes between wild‐type and R5‐mutant.

	Δ*H*‡ [kJ/mol]	ΔΔ*H*‡ [kJ/mol]	Δ*H* [kJ/mol]	ΔΔH [kJ/mol]
Wild‐type	16	‐	−89	‐
R5‐mutant	−55	−71	−120	−31

Because previous studies suggested that improving antibody affinity toward antigen can lead to destabilization of the antibody, we evaluated the thermal stability of the R5‐mutant by differential scanning calorimetry (DSC) analysis. The Tm values of wild‐type and R5‐mutant were 78.3°C and 75.6°C, respectively (Figure S2), indicating that the R5 mutation indeed destabilized the Fab.

### 2.2 Structure of the antibody–antigen complex of the R5‐mutant

**FIGURE 2:**
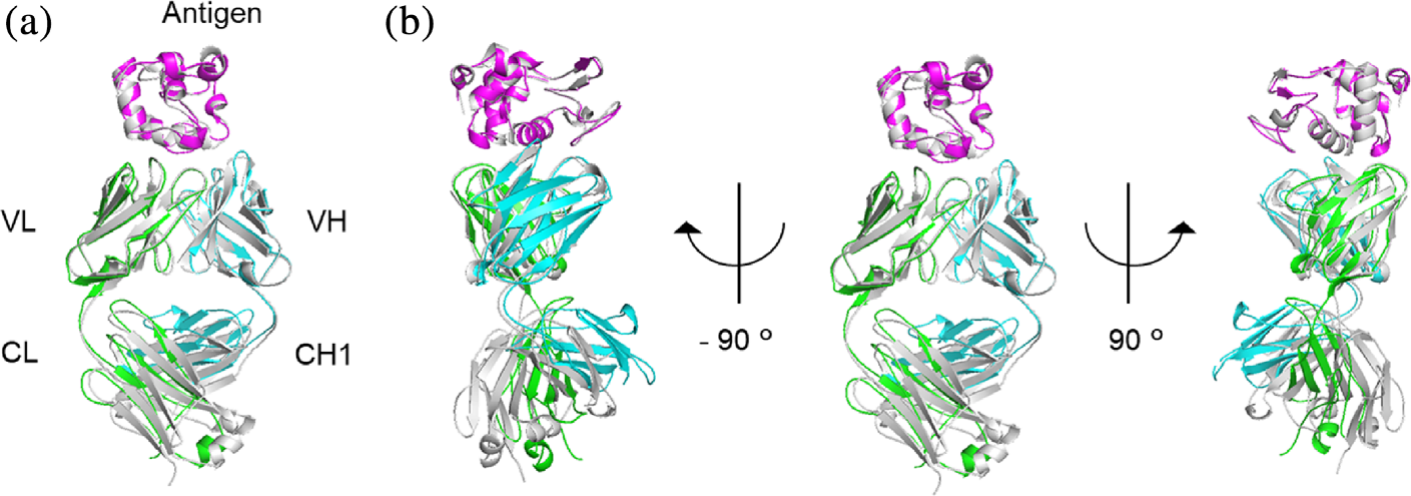
Structure of the HyHEL‐10 Fab. (a) X‐ray crystal structure of the R5‐mutant in complex with antigen. (b) Structural superposition of the Fab antigen complex keeping the fixed antigen. The wild‐type HyHEL‐10 (PDB ID: 3D9A) is depicted in gray. Magenta, blue, and green show antigen, light chain, and heavy chain, respectively, in the R5‐mutant complex. Structures were generated using PyMOL (ver. 2.5.4).

To identify the molecular mechanism by which the R5‐mutant gained enhanced affinity, we conducted structural analysis and determined the crystal structure of the R5‐mutant antigen complex at a resolution of 3.0 Å. Although some residues in the CH1 region (Gly 127–Gln 133) were not modeled because of missing or weak electron density, the entire structure exhibited typical Fab and antigen complex geometry (Figure 2a).

**FIGURE 3:**
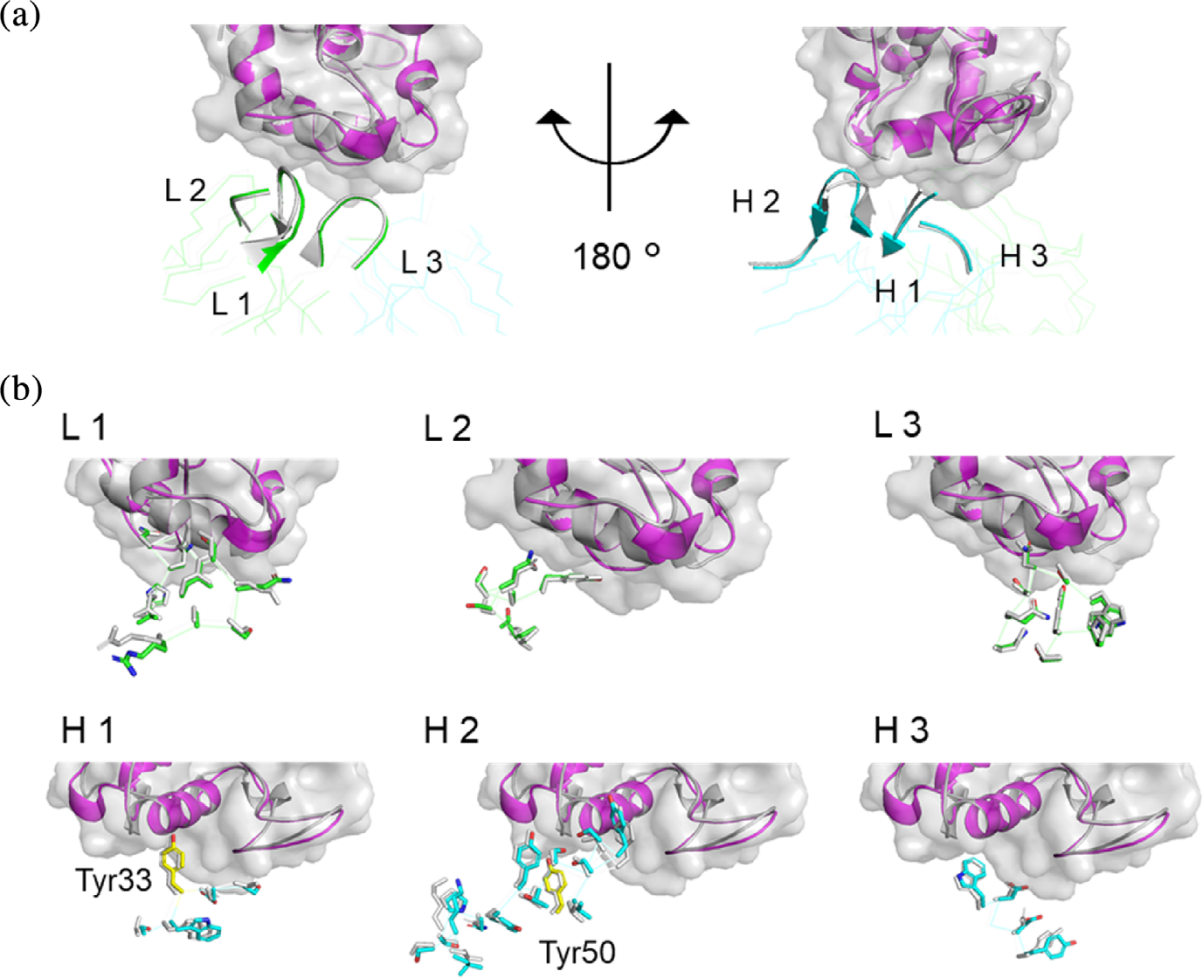
Structural comparison between wild‐type and R5‐mutant. Superposition of CDR loops (L1–H3) are shown in (a) ribbon diagram and (b) side chain. Wild‐type HyHEL‐10 (PDB ID: 3D9A) is shown in gray. Magenta, blue, and green show antigen, light chain, and heavy chain, respectively, in the R5‐mutant.

To compare the overall structure of the R5‐mutant‐antigen complex with that of the wild‐type‐antigen complex, we superposed the crystal structure of the former onto that of the latter (PDB ID: 3D9A) by fitting Cα atoms of the antigen. The overall structure within FR3 was very similar between wild‐type and R5‐mutant complexes. We calculated the RMSD values of the Cα for each domain after superposing the particular domain (Table 3). The orientation of Fab and antigen was almost identical for the wild‐type and R5‐mutant (Figure 2b). The relative position of variable and constant regions differed slightly between the wild‐type and R5‐mutant. This difference was caused by the change in the elbow angle, defined as the angle between the pseudo‐dyad axes relating VL to VH and CL to CH1 (Stanfield et al., 2006), that occurred from 148° to 122°. Because previous studies showed that the available elbow angles cover a wide range (127° to 220°) (Stanfield et al., 2006) and both wild‐type and R5‐mutant elbow angles were close to this range, the structure of the Fab and antigen complex did not change substantially after introducing arginine residues. Furthermore, orientations of side chains in each CDR loop, including Tyr 33 in CDR H1 and Tyr 50 in CDR H2, which are identified as hot spot residues (Shiroishi et al., 2007), were not changed by the mutation (Figure 3).

**TABLE 3:** RMSD values of the Cα for each domain after superposing the particular domain.

RMSD (Å) superpose	Antigen	VH	VL	CH1	CL
Antigen	0.61	1.2	1.2	3.3	3.5
VH	0.99	0.64	1.3	2.8	3.4
VL	1.5	1.9	0.45	2.6	3.3
CH1	2.7	3.2	2.8	0.70	0.92
CL	3.4	3.1	2.7	0.98	0.74

*Note*: VH, VL and CH1, CL corresponds to variable region of heavy chain and light chain and constant region of heavy chain and light chain, respectively.

#### 2.2.1 Structural dynamics of the R5‐mutant determined using MD simulations

To gain an insight into the molecular mechanism which improve the affinity of the antibody, we conducted MD simulations to evaluate the dynamics of both wild‐type and R5‐mutant in the unbound state. We computed the RMSD of the Cα atoms (Figure S3a) and found that the values were quite stable after 50 ns. Therefore, in the analyses below, we did not consider the first 50 ns of the trajectories.

First, we computed the Cα‐RMSD of light chain FR3, which contains mutation points, after superposing Cα atoms of the variable region excluding FR3 to evaluate the effect of R5 mutation on the surrounding region. The RMSD of the R5‐mutant was stable and similar to that of the wild‐type over the course of the 400 ns simulation time (Figure S3b). This result indicated no significant changes in this region due to arginine introduction.

**FIGURE 4:**
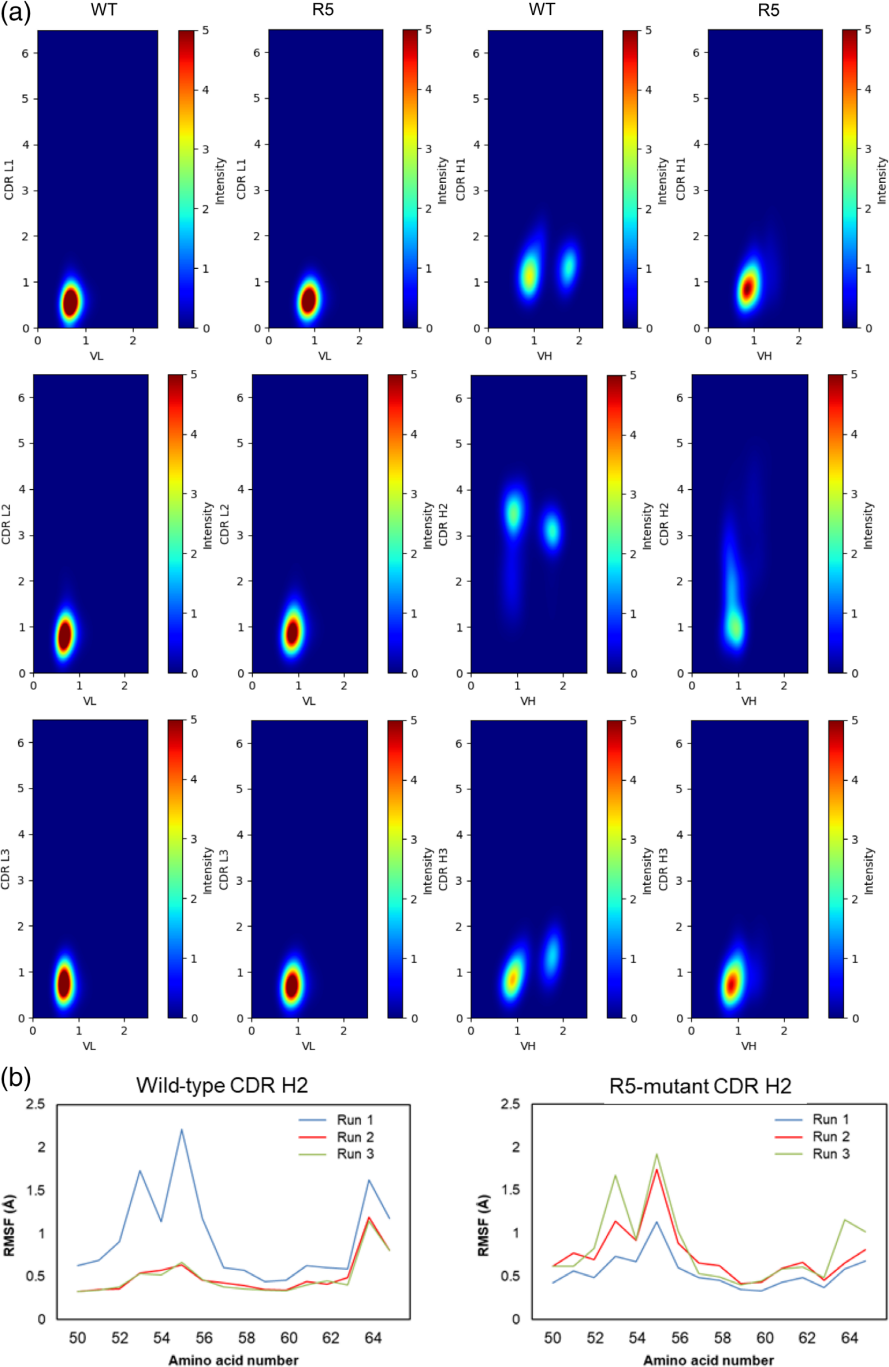
Comparison of MD simulation trajectories. RMSDs of the Cα atoms between 400 ns simulations and the initial structure. (a) 2D‐RMSD plots of Cα‐RMSDs for each individual loop versus variable regions containing respective CDRs. The last 350 ns trajectories of three simulations were combined in one plot. (b) Root mean square fluctuation difference in CDR H2.

Because the affinity of antibodies toward antigens depends greatly on CDR flexibility (Jeliazkov et al., 2018; Ovchinnikov et al., 2018). we next focused on the dynamics of six CDR loops. When we superposed Cα atoms of the variable region of the light chain or heavy chain and computed RMSDs of Cα for each CDR loop, we observed a different RMSD pattern only in the heavy chain CDR 2 region (CDR H2), whereas the other CDR loops showed a similar tendency between the wild‐type and R5‐mutant over the course of the simulation time (Figure S3c). For CDR H2, the RMSD of the R5‐mutant was distributed more widely compared to the wild‐type (Figure S3d). To further examine the conformational change in CDR loops, we created 2D‐RMSD plots. In these plots, two RMSD values are represented on the *X*‐axis and *Y*‐axis respectively, offering a visual depiction of conformational space. We combined all data from the 350 ns simulation into a single plot for each individual loop (Figure 4a). Looking at the heavy chain plots, while there are two separate spots in WT, only one spot was observed in R5 mutant. Also, the RMSD values for CDR H2 largely varied between WT and R5 mutant. Whereas the RMSD value for CDR H2 in WT converged at around 3 Å, the RMSD value in R5 mutant broadly scattered between 1 and 2 Å with dense spot at around 1 Å. Furthermore, we conducted a principal component analysis to compare the dynamics of each CDR loop in WT and R5 mutant and the results are shown in Figure S4. While the distribution of plots against PC1 and PC2 shows similar tendency for each CDR except CDR H2, that for CDR H2 significantly changed (Figure S4). Collectively, these results suggest that the R5 mutant exhibits conformational diversity, which would be closer to the conformation in its bound state than WT. We also calculated the root mean square fluctuation for each residue in CDR H2 and found higher fluctuation in the R5‐mutant compared to the wild‐type, especially in the region from amino acid numbers 50 to 58 (Figure 4b).

**FIGURE 5:**
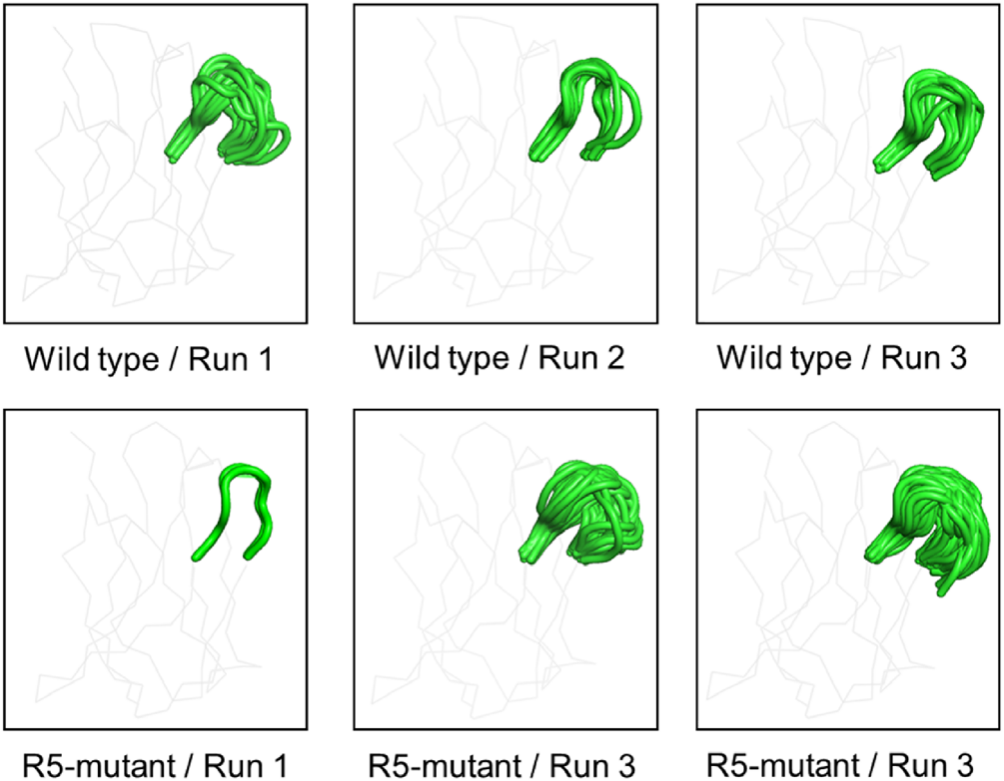
Average structure of the CDR H2 loop (amino acid numbers 50–58) for each cluster. Single linkage method was used to calculate clusters in each 400 ns simulations. The CDR H2 loop is shown in red.

**FIGURE 6:**
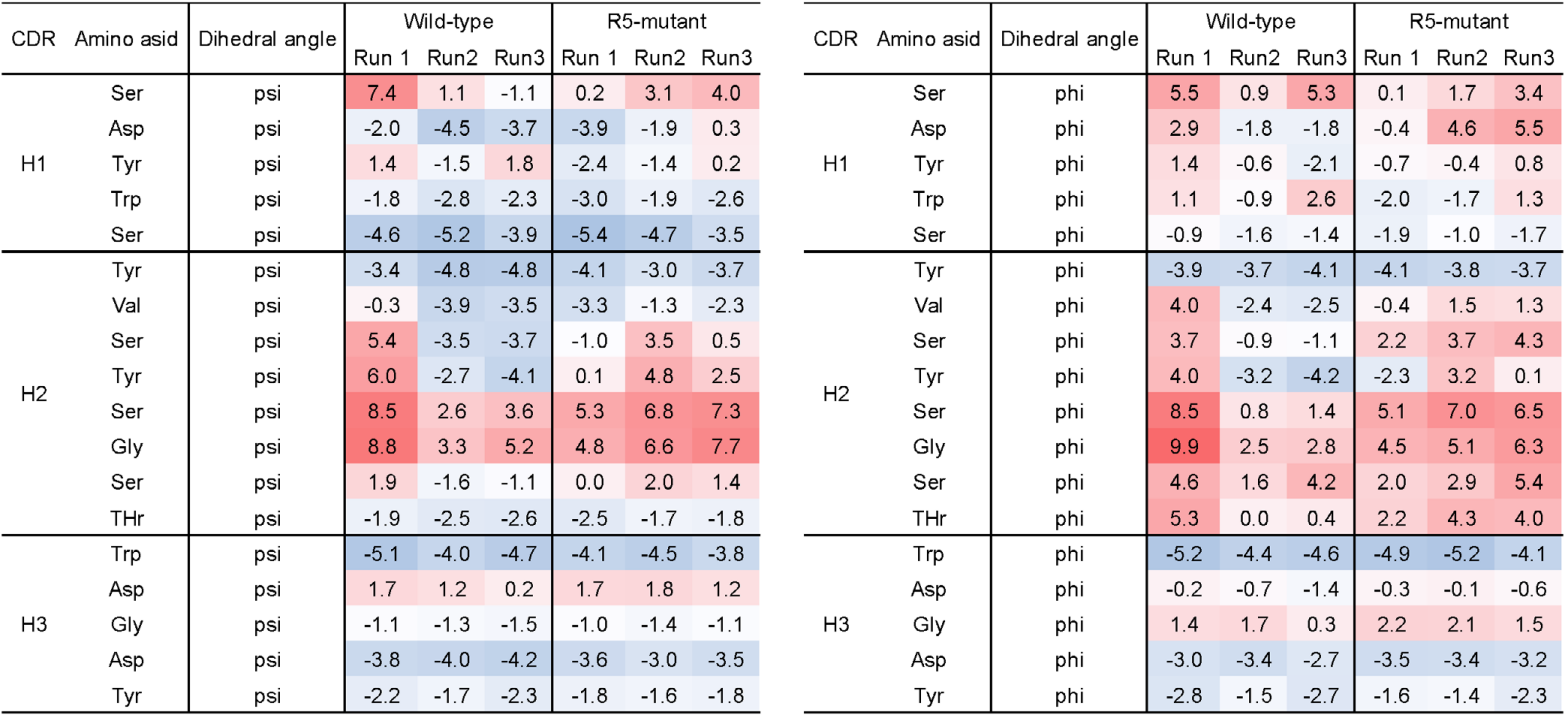
Dihedral entropy values of the heavy chain CDR loop. Dihedral angles were calculated for each phi and psi in CDR H2, and dihedral entropy was computed.

We evaluated the structural diversity of CDR H2 by clustering CDR H2 structures with a 0.5 Å cut‐off using the single linkage method as implemented in Gromacs (Hess et al., 2008). Number of clusters are summarized in Table 4 and average structures of the CDR H2 loop for each cluster were shown in Figure 5. Among three runs for R5‐mutant, we observed much less cluster only for run1. This may be due to the existence of a local minima in which simulation run1 for R5‐mutant would be trapped. Meanwhile, looking at the clustering of loop structures in WT, the number of clusters was stable and less than that of the R5‐mutant, except run1 of R5‐mutant. Therefore, as a whole, we observed that the R5‐mutant had higher conformational diversity than the wild‐type. To ensure the observed differences in fluctuation did not stem from insufficient convergence, we divided the trajectories from each run into two sections, 50 to 225 ns and 225 to 400 ns, and compared the RMSF derived from each (Figure S5). The plots overlapped noticeably, even between different runs, suggesting that our trajectories have converged well. We then analyzed the flexibility of each CDR loop using dihedral entropies (Kraml et al., 2021). The results demonstrated that the dihedral entropy values increased for the residues in CDR H2 as a result of the R5 mutation (Figure 6). Collectively, these results suggest that R5 mutation induced more conformational variations in the CDR H2 loop, which increased the flexibility of the loop and thereby increased the affinity for antigen.

**TABLE 4:** Number of clusters in the CDR H2 loop (amino acid numbers 50–58) for the 400 ns simulation for the wild‐type and R5‐mutant.

	Run	Number of clusters
Wild‐type	1	21
	2	9
	3	20
R5‐mutant	1	2
	2	49
	3	100

## 3 DISCUSSION

In this study, we conducted SPR analysis using the wild‐type and R5‐mutant of the HyHEL10 Fab and determined kinetic parameters of the interaction of the Fabs with the antigen. Kinetic analysis showed that the R5‐mutant had an increased *k*_on_ value and higher affinity compared to the wild‐type. In the process of interaction, antigen and antibody initially form an encounter complex with opposite charges, and the electrostatic interaction at a long distance caused by the surface charge improves the collision frequency (Northrup and Erickson, 1992). However, given that hen egg white lysozyme (HEL), the antigen of HyHEL‐10, has a high content of basic amino acids and thus its isoelectric point is calculated to be 11.0 (Carrillo et al., 2016). On the other hand, the isoelectric point of the wild‐type and R5‐mutant, calculated by Discovery Studio (ver. 20.1; BIOVIA), was 7.6 and 9.6, respectively. Thus, it is not likely that the greater *k*_on_ value of the R5‐mutant was derived from the simple electrostatic interaction.

In the present study, the crystal structure of the Fab‐antigen complex showed that the orientation of Fab and antigen was almost identical between wild‐type and R5‐mutant, indicating that the R5‐mutant maintained the antigen epitope after introduction of the arginine mutation. Yili et al. previously suggested that antibody maturation improves the binding affinity through burial of hydrophobic surfaces at the periphery of the interface of antigen binding, resulting in rearrangements in non‐hot spot residues (Li et al., 2003). Thus, the molecular mechanism underlying affinity improvement caused by the R5‐mutation is likely completely different from the natural affinity maturation process. Considering that the R5‐mutation decreased thermal stability, applying other antibody stabilizing technologies (Julian et al., 2017; Gong et al., 2009) together with our R5‐mutation may effectively improve antibody function.

The result of MD simulations suggested that the R5‐mutant had increased flexibility in CDR H2. Our SPR analysis showing rapid association and lower enthalpy change in the transition state together with the DSC results suggest that this increased flexibility would raise the energy state in the unbound form, resulting in a lower energy barrier in the transition state during the interaction steps. Kiyoshi et al. previously showed that it is important to reduce this energy barrier, which can be evaluated by analyzing the enthalpy change (Δ*H*‡), to improvement affinity (Kiyoshi et al., 2014). We found that the R5‐mutant in our study improved the *k*_on_ value by reducing the energy barrier to the transition state due to the increased flexibility of the CDR H2 loop. Generally, the increase in flexibility in apo‐form would result in the increase of entropy loss during interaction process and lead to lower binding affinity. However, considering the larger favorable enthalpic change for R5‐mutant than wild‐type in transition state (Table 2), our R5 mutation should significantly influence on the encounter complex formation process by facilitating the non‐covalent bonds constitution during the process, and thereby accelerate the interaction, leading to faster association. Taken together with the crystal structure of the complex in which the structure of CDRH2 was not significantly changed by R5 mutation, the dynamic structural changes observed in the MD simulation would result in the faster binding rate constants. Given that the increased fluctuation in CDR of heavy chain was induced by the arginine cluster introduction into the framework region in the light chain, it is likely that the modification caused some changes in dynamics of surrounding framework region, then it transmitted to the CDR through VH–VL interface. As previous studies showed this VH–VL relative orientation impacts on the CDR loop states and thereby affect the antigen binding (Fernández‐Quintero et al., 2020), and also showed that framework residues influence dynamic structure of CDR (Kelow et al., 2020; Fernández‐Quintero et al., 2021), the arginine cluster introduction would adopt similar mechanism to enhance the binding activity.

Our MD simulation results indicate that all CDR loops except CDR H2 from both wild‐type and R5‐mutant did not have large conformational variations in the un‐bound state, whereas the CDR H2 loop underwent more conformational changes. Although the wild‐type also had certain degree of flexibility in CDR H2, the R5‐mutation had enhanced flexibility, resulting in a greater number of conformational clusters. The underlying mechanism of antibody–antigen complex formation has several phases (Horn et al., 2009). Antigen and antibody initially collide with each other, bringing a potential epitope in proximity to an antibody CDR, and they then must proceed through a free energy maximum (the transition state) to reach the bound (antigen–antibody complex) state. Before reaching the bound state, antibody and antigen first form a weakly interacting complex in which the hydration of the protein surface is not altered significantly. During this process, the structure is rearranged so that the CDRs and epitopes are placed in the proper orientation and exploring multiple candidate structures to make a complex with antigen. Therefore, increased flexibility of CDRs caused by the R5 mutation would induce a variety of structural ensembles, which in turn would generate structures suitable for making a complex and thus contribute to faster association.

In conclusion, our results suggest that introducing the R5 mutation into the framework region of antibodies does not alter the overall complex structure, but it increases the conformational diversity of the CDR, thereby enhancing the antibody–antigen association rate. Because the strategy does not require detailed characterization of the interaction between antibody and antigen in advance, our strategy may be easily applicable to a variety of antibodies and lead to a general strategy for antibody engineering.

## 4 METHODS

### 4.1 Preparation of HyHEL10 Fab fragments

Gene fragments encoding heavy and light chains containing the R5 mutation were cloned into pcDNA 3.4, an expression vector for mammalian expression systems (Thermo Fisher Scientific) with a signal peptide sequence. Expi293 cells (Thermo Fisher Scientific) were co‐transfected with expression vectors, and supernatant was collected 4 days after transfection. The supernatant was dialyzed against 20 mM Tris–HCl (pH 8.0), 500 mM NaCl 5 mM imidazole (biding buffer) and loaded on Ni‐NTA resin (QIAGEN) equilibrated with the binding buffer. The resin was washed with 20 mM Tris–HCl (pH 8.0) 500 mM NaCl 20 mM imidazole (wash buffer), and subsequently Fabs were eluted by 20 mM Tris–HCl (pH 8.0), 500 mM NaCl, 500 mM imidazole. Hen egg white lysozyme was purchased from commercial sources (Sigma‐Aldrich: L4919).

### 4.2 Binding analysis of the HyHEL‐10 Fab using surface plasmon resonance

We prepared a HyHEL‐10 Fab mutant in which five arginine residues were introduced into framework region 3 (FR3) (R5‐mutant: LS63R, LS65R, LS67R, LD70R, LT72R). SPR experiments were performed using a BIAcoreT200 system (Cytiva) as previously described (Löhr et al., 2022). Briefly, hen egg white lysozyme (Sigma‐Aldrich) was immobilized on research‐grade CM5 sensor chips (Cytiva). The purified wild‐type and R5‐mutant were dialyzed against HBS‐EP buffer (10 mM HEPES [pH 7.4], 150 mM NaCl, 3.4 mM EDTA, 0.05% surfactant P20) and injected over the immobilized antigen at a flow rate of 30 μL/min. We used BIA evaluation software version 2.0.2 (Cytiva) to analyze the data. The association and dissociation rate constants (*k*_on_ and *k*_off_) were determined by a global fitting analysis. The dissociation constant Kd was also calculated from the value of *k*_on_ and *k*_off_. Thermodynamic analyses of each Fab were performed at four temperature points (302.15, 306.15, 310.15, and 314.15 K).

### 4.3 Differential scanning calorimetry measurements

DSC thermograms were monitored from 40 to 90°C to evaluate the melting temperature (Tm) using a VP‐DSC MicroCalorimeter (Malvern). The antibodies were diluted to 5 μM with 10 mM HEPES, 150 mM NaCl at pH 7.3 before loading into the sample cell. The heating rate was set to 1°C/min. A thermogram of the reference buffer was also obtained, and sample thermograms were normalized by subtracting the response of the reference buffer. We used Origin 7.0 software for data analysis (OriginLab Corporation).

### 4.4 Crystallization and X‐ray crystallography data collection/refinement

The Fabs were dialyzed against 10 mM Tris–HCl (pH 8.0), 150 mM NaCl together with lysozyme and further purified by size exclusion chromatography using a HiLoad 26/600 Superdex 75‐pg column (Cytiva) equilibrated with 10 mM Tris–HCl (pH 8.0), 150 mM NaCl. The crystallization screening was carried out using an Oryx8 protein crystallization robot (Douglas Instruments). Small‐sized crystals were obtained in 0.2 M sodium citrate tribasic dihydrate, 0.1 M Tris hydrochloride (pH 8.5), 30% polyethylene glycol 400. The obtained crystals were harvested with conventional cryoloops and cryocooled in liquid nitrogen. Diffraction data were collected using the automated data collection system, *ZOO* (Hirata et al., 2019), at the beamline BL32XU of SPring‐8 in Japan. The diffraction data from 21 crystals were merged, and finally obtained the data with a resolution of 3.13 Å. Molecular replacement was performed using *CCP4 PHASER* (McCoy et al., 2007) using the obtained structure factor and a model with a similar structure (PDB ID: 3D9A). After the molecular replacement, 100 cycles of jelly body refinement was performed using *CCP4 REFMAC5* (Murshudov et al., 2011). Several iterations of manual model building using *Coot* (Emsley et al., 2010) and 30 cycles of jelly‐body refinement were carried out followed by the restrained refinement using *phenix.refine* (Liebschner et al., 2019) to obtain the final structure. The *R*_work_/*R*_free_ factors of the final model were 0.231 and 0.269, respectively. Data collection and refinement statistics are summarized in Table 5 and the final model was deposited to Protein Data Bank as PDB ID: 8GQ1.

**TABLE 5:** Data collection and refinement statistics.

	HyHEL10‐R5 variant
Data collection	
Wavelength [Å]	1.0000
Resolution range [Å]	19.66–3.13 (3.24–3.13)
Space group	*P*6_3_22
Unit cell constants	
*a*, *b*, *c* [Å]	147.9, 147.9, 137.7
*α*, *β*, *γ* [°]	90, 90, 120
Total reflections	32,247 (3132)
Unique reflections	16,124 (1566)
Redundancy	2.0 (2.0)
Completeness [%]	99.35 (99.94)
<I/σI>	8.86 (1.08)
Rmerge	0.090 (0.575)
Rmeas	0.128 (0.813)
CC1/2	0.987 (0.545)
Refinement	
Reflections used in refinement	16,118 (1565)
Reflections used for *R*_free_	806 (78)
*R*_work_	0.2311 (0.3179)
*R*_free_	0.2693 (0.3029)
Number of non‐hydrogen atoms	4191
Macromolecules	4175
Ligands	16
Solvent	0
RMSD	
Bond length	0.005
Angles	0.88
Ramachandran plot	
Favored [%]	98.30
Allowed [%]	1.70
Outliers [%]	0.00
Rotamer outliers [%]	0.42
Clashscore	5.84
*B*‐factor	
Average	66.54
Macromolecules	66.58
Ligands	55.84

### 4.5 MD simulation

MD simulation was performed using GROMACS 2016.3 with the CHARMM 36 m force field. The Fab structure of the HyHel10 Fab/HEL complex (PDB ID: 3D9A) was used as the wild‐type structure. For the R5‐mutant structure, the mutated residues were substituted for the corresponding wild‐type residue. Using the CHARMM GUI, both wild‐type and R5‐mutant structures were solvated with TIP3P water in a rectangular box such that the minimum distance to the edge of the box was 15 Å under periodic boundary conditions. Na and Cl ions were added to neutralize the protein charge, then further ions were added to mimic a salt solution concentration of 0.15 M. Each system was energy minimized for 10,000 steps and equilibrated with NVT ensemble at 298 K for 1 ns. A further production run was performed for 400 ns with NPT ensemble, and the time step was set to 2 fs throughout the simulations. A simulation was repeated three times for both systems of wild‐type and R5‐mutant with different initial velocities, and the snapshots were saved every 10 ps. To assess the stability of our simulations, we first computed the root mean‐square deviation (RMSD) of the Cα atoms after superimposing the Cα atoms of each structure during the simulations. Reference to the initial structure of the production runs suggested that our simulations were well equilibrated after 50 ns, therefore the last 350 ns trajectories of each simulation was used for analysis. Principal component analysis was performed using analysis modules implemented in Gromacs. Principal component analysis was performed on MD trajectories using Cα atoms. The trajectories were processed using the Gromacs tool gmx covar, and covariance matrices were computed for each CDR loop after superposing with the FR. The obtained covariance matrix was analyzed with the gmx anaeig command to obtain eigenvectors. The results were visualized by matplotlib library in python (Hunter, 2007). To evaluate the flexibility of the CDR loops, phi and psi dihedral entropies were computed with the X‐Entropy package (Kraml et al., 2021). Both phi and psi dihedral angles were calculated with the gmx chi command of gromacs.

## AUTHOR CONTRIBUTIONS

**Shingo Maeta:** Conceptualization (equal); data curation (lead); formal analysis (equal); investigation (lead); writing – original draft (lead). **Makoto Nakakido:** Conceptualization (equal); funding acquisition (equal); investigation (equal); project administration (equal); writing – original draft (equal). **Hiroaki Matsuura:** Data curation (equal); formal analysis (equal); investigation (equal); writing – review and editing (equal). **Naoki Sakai:** Data curation (supporting); investigation (supporting); resources (equal); software (equal); writing – review and editing (supporting). **Kunio Hirata:** Data curation (equal); investigation (supporting); project administration (supporting); resources (equal); software (equal); supervision (equal); writing – review and editing (supporting). **Daisuke Kuroda:** Conceptualization (equal); data curation (supporting); investigation (supporting); methodology (equal); software (equal); supervision (equal); writing – review and editing (equal). **Atsushi Fukunaga:** Conceptualization (lead); data curation (supporting); project administration (equal); resources (equal); supervision (lead); writing – review and editing (supporting). **Kohei Tsumoto:** Conceptualization (equal); data curation (supporting); funding acquisition (lead); project administration (lead); resources (equal); supervision (lead); writing – review and editing (equal).

## FUNDING INFORMATION

This work was funded in part by Japan Society for the Promotion of Science (grant no. 21H05090 to Makoto Nakakido, 19H05766 and 20H02531 to Kouhei Tsumoto), by JST‐Mirai Program grant number JP MJMI21G6 to Makoto Nakakido, JST‐CREST Program Grant Number JP MJCR20H8 to Kouhei Tsumoto, and Japan Agency for Medical Research and Development (grant no. JP 18ak0101100 and 19am0401010to Makoto Nakakido, 20mk0101170 to Kouhei Tsumoto).

## CONFLICT OF INTEREST STATEMENT

Shingo Maeta and Atsushi Fukunaga are employed by Sysmex corporation. A patent application has been filed relating to this work.

## Supporting information


**FIGURE S1:** SPR sensorgrams for mutants with mutation in the hot‐spot residue. (a) HY33F and HY50F. (b) Additional 5‐points Arg mutation HY33F‐R5 and HY50F‐R5. (c) Alanine mutants of the hot‐spot (HY33A and HY50A) and additional 5‐points Arg mutation (HY33A‐R5 and HY50A‐R5). Running buffer injection was subtracted as the blank (gray: raw data; black: fitting data).
**FIGURE S2.** The figure contains DSC thermogram and Tm values calculated from the analysis. R5‐mutant has a peak at lower temperature compared to wild‐type and the6 calculated Tm values were 78.3°C for wild‐type and 75.6°C for R5‐mutant, indicating the R5 mutation destabilized the Fab.
**FIGURE S3.** Comparison of MD simulation trajectories. RMSDs of the Cα atoms between 400 ns simulations and the initial structure. Each 400 ns run was performed three times, indicated by blue, red, and green lines. Averages and standard deviations for each run are shown in the figure. Calculated in (a) total Fab molecule and (b) framework region 3 and (c) each CDR loop. (d) The distribution of the RMSD of CDR H2.
**FIGURE S4.** Principal component analysis of each CDR loop in wild‐type and R5‐mutant.
**FIGURE S5.** RMSF comparison with trajectory splitting. RMSF values calculated from each trajectory were overlapped. Trajectory was split into first 175 ns and last 175 ns for each simulation run.
**TABLE S1.** Kinetic parameters for mutants of hot‐spot residue.

## References

[pro4745-bib-0001] Adair JR . Engineering antibodies for therapy. Immunol Rev. 1992;130:5–40. 1286872 10.1111/j.1600-065x.1992.tb01519.x

[pro4745-bib-0002] Adams GP , Schier R , Marshall K , Wolf EJ , Mccall AM , Marks JD , et al. Increased affinity leads to improved selective tumor delivery of single‐chain Fv antibodies. Cancer Res. 1998;58:485–490. 9458094

[pro4745-bib-0003] Akiba H , Tamura H , Kiyoshi M , Yanaka S , Sugase K , Caaveiro JMM , et al. Structural and thermodynamic basis for the recognition of the substratebinding cleft on hen egg lysozyme by a single‐domain antibody. Sci Rep. 2019;9:15481. 31664051 10.1038/s41598-019-50722-y PMC6820745

[pro4745-bib-0004] Carrillo W , Tubón J , Vilcacundo R . Isolation of hen egg white lysozyme by cation exchange chromatography, analysis of its digestibility and evaluation of the inhibition lipid peroxidation in the zebrafish model. Asian J Pharm Clin Res. 2016;9:345–349.

[pro4745-bib-0005] Emsley P , Lohkamp B , Scott WG , Cowtan K . Features and development of Coot. Acta Crystallogr Sect D Biol Crystallogr. 2010;66(Pt4):486–501. 20383002 10.1107/S0907444910007493 PMC2852313

[pro4745-bib-0006] Fernández‐Quintero ML , Georges G , Varga JM , Liedl KR . Ensembles in solution as a new paradigm for antibody structure prediction and design. MAbs. 2021;13(1):1923122. 34030577 10.1080/19420862.2021.1923122 PMC8158028

[pro4745-bib-0007] Fernández‐Quintero ML , Kroell KB , Hofer F , Riccabona JR , Liedl KR . Mutation of framework residue H71 results in different antibody paratope states in solution. Front Immunol. 2021;12:243. 10.3389/fimmu.2021.630034 PMC7960778 33737932

[pro4745-bib-0008] Fernández‐Quintero ML , Loeffler JR , Bacher LM , Waibl F , Seidler CA , Liedl KR . Local and global rigidification upon antibody affinity maturation. Front Mol Biosci. 2020;7:182. 32850970 10.3389/fmolb.2020.00182 PMC7426445

[pro4745-bib-0009] Fernández‐Quintero ML , Pomarici ND , Math BA , Kroell KB , Waibl F , Bujotzek A , et al. Antibodies exhibit multiple paratope states influencing VH–VL domain orientations. Commun Biol. 2020;3(1):589. 33082531 10.1038/s42003-020-01319-z PMC7576833

[pro4745-bib-0010] Fukunaga A , Maeta S , Reema B , Nakakido M , Tsumoto K . Improvement of antibody affinity by introduction of basic amino acid residues into the framework region. Biochem Biophys Rep. 2018;15:81–85. 30073208 10.1016/j.bbrep.2018.07.005 PMC6068084

[pro4745-bib-0011] Fukunaga A , Tsumoto K . Improving the affinity of an antibody for its antigen via long‐range electrostatic interactions. Protein Eng Des Sel. 2013;26:773–780. 24214686 10.1093/protein/gzt053

[pro4745-bib-0012] Gong R , Vu BK , Feng Y , Prieto DRA , Dyba MA , Walsh JD , et al. Engineered human antibody constant domains with increased stability. J Biol Chem. 2009;284:14203–14210. 19307178 10.1074/jbc.M900769200 PMC2682868

[pro4745-bib-0013] Hess B , Kutzner C , Van Der Spoel D , Lindahl E . GROMACS 4: algorithms for highly efficient, load‐balanced, and scalable molecular simulation. J Chem Theory Comput. 2008;4:435–447. 26620784 10.1021/ct700301q

[pro4745-bib-0014] Hirata K , Yamashita K , Ueno G , Kawano Y , Hasegawa K , Kumasaka T , et al. Zoo: an automatic data‐collection system for high‐throughput structure analysis in protein microcrystallography. Acta Crystallogr Sect D Struct Biol. 2019;75:138–150. 30821703 10.1107/S2059798318017795 PMC6400253

[pro4745-bib-0015] Horn JR , Sosnick TR , Kossiakoff A . Principal determinants leading to transition state formation of a protein–protein complex, orientation trumps sidechain interactions. Proc Natl Acad Sci U S A. 2009;106:2559–2564. 19196954 10.1073/pnas.0809800106 PMC2650303

[pro4745-bib-0016] Hunter DJ . matplotlib: a 2D graphics environment. Comput Sci Eng. 2007;9(3):90–95.

[pro4745-bib-0017] Jeliazkov JR , Sljoka A , Kuroda D , Tsuchimura N , Katoh N , Tsumoto K , et al. Repertoire analysis of antibody CDR‐H3 loops suggests affinity maturation does not typically result in rigidification. Front Immunol. 2018;9:413. 29545810 10.3389/fimmu.2018.00413 PMC5840193

[pro4745-bib-0018] Julian MC , Li L , Garde S , Wilen R , Tessier MP . Efficient affinity maturation of antibody variable domains requires co‐selection of compensatory mutations to maintain thermodynamic stability. Sci Rep. 2017;7:45259. 28349921 10.1038/srep45259 PMC5368667

[pro4745-bib-0019] Kabat EA , Wu TT , Bilofsky H . Some correlations between specificity and sequence of the first complementarity‐determining segments of human kappa light chains. Proc Natl Acad Sci U S A. 1976;73:4471–4473. 826908 10.1073/pnas.73.12.4471 PMC431504

[pro4745-bib-0020] Kabat EA , Wu TT , Bilofsky H . Unusual distributions of amino acids in complementarity‐determining (hypervariable) segments of heavy and light chains of immunoglobulins and their possible roles in specificity of antibody‐combining sites. J Biol Chem. 1977;252:6609–6616. 408353

[pro4745-bib-0021] Kelow SP , Adolf‐Bryfogle J , Dunbrack RL . Hiding in plain sight: structure and sequence analysis reveals the importance of the antibody DE loop for antibody‐antigen binding. MAbs. 2020;12(1):1840005. 33180672 10.1080/19420862.2020.1840005 PMC7671036

[pro4745-bib-0022] Kempeni J . Preliminary results of early clinical trials with the fully human antiTNFα monoclonal antibody D2E7. Ann Rheum Dis. 1999;58:170–i72. 10.1136/ard.58.2008.i70 PMC1766582 10577977

[pro4745-bib-0023] Kirsch MI , Hülseweh B , Nacke C , Rülker T , Schirrmann T , Marschall HJ , et al. Development of human antibody fragments using antibody phage display for the detection and diagnosis of Venezuelan equine encephalitis virus (VEEV). BMC Biotechnol. 2008;8:1–15. 18764933 10.1186/1472-6750-8-66 PMC2543005

[pro4745-bib-0024] Kiyoshi M , Caaveiro JMM , Miura E , Nagatoishi S , Nakakido M , Soga S , et al. Affinity improvement of a therapeutic antibody by structure‐based computational design: generation of electrostatic interactions in the transition state stabilizes the antibody‐antigen complex. PLoS One. 2014;9(1):e87099. 24475232 10.1371/journal.pone.0087099 PMC3903617

[pro4745-bib-0025] Kraml J , Hofer F , Quoika PK , Kamenik AS , Liedl KR . X‐entropy: a parallelized kernel density estimator with automated bandwidth selection to calculate entropy. J Chem Inf Model. 2021;61:1533–1538. 33719418 10.1021/acs.jcim.0c01375 PMC8154256

[pro4745-bib-0026] Lawson ADG . Antibody‐enabled small‐molecule drug discovery. Nat Rev Drug Discov. 2012;11:519–525. 22743979 10.1038/nrd3756

[pro4745-bib-0027] Li Y , Li H , Yang F , Smith‐Gill SJ , Mariuzza AR . X‐ray snapshots of the maturation of an antibody response to a protein antigen. Nat Struct Biol. 2003;10:482–488. 12740607 10.1038/nsb930

[pro4745-bib-0028] Liebschner D , Afonine PV , Baker ML , Bunkoczi G , Chen VB , Croll TI , et al. Macromolecular structure determination using X‐rays, neutrons and electrons: recent developments in phenix. Acta Crystallogr Sect D Struct Biol. 2019;75:861–877. 31588918 10.1107/S2059798319011471 PMC6778852

[pro4745-bib-0029] Löhr T , Sormanni P , Vendruscolo M . Conformational entropy as a potential liability of computationally designed antibodies. Biomolecules. 2022;12:718. 35625644 10.3390/biom12050718 PMC9138470

[pro4745-bib-0030] McCoy AJ , Grosse‐Kunstleve RW , Adams PD , Winn MD , Storoni LC , Read JR . Phaser crystallographic software. J Appl Cryst. 2007;40:658–674. 19461840 10.1107/S0021889807021206 PMC2483472

[pro4745-bib-0031] Muda M , Gross AW , Dawson JP , He C , Kurosawa E , Schweickhardt R , et al. Therapeutic assessment of SEED: a new engineered antibody platform designed to generate mono‐ and bispecific antibodies. Protein Eng Des Sel. 2011;24:447–454. 21498564 10.1093/protein/gzq123

[pro4745-bib-0032] Murshudov GN , Skubák P , Lebedev AA , Pannu NS , Steiner RA , Nicholls RA , et al. REFMAC5 for the refinement of macromolecular crystal structures. Acta Crystallogr Sect D Struct Biol. 2011;67:355–367. 10.1107/S0907444911001314 PMC3069751 21460454

[pro4745-bib-0033] Northrup SH , Erickson H . Kinetics of protein‐protein association explained by Brownian dynamics computer simulation. Proc Natl Acad Sci U S A. 1992;89:3338–3342. 1565624 10.1073/pnas.89.8.3338 PMC48862

[pro4745-bib-0034] Ovchinnikov V , Louveau JE , Barton JP , Karplus M , Chakraborty KA . Role of framework mutations and antibody flexibility in the evolution of broadly neutralizing antibodies. Elife. 2018;14:e3308. 10.7554/eLife.33038 PMC5828663 29442996

[pro4745-bib-0035] Roovers RC , Henderikx P , Helfrich W , Van Der Linden E , Reurs A , De Bruine AP , et al. High‐affinity recombinant phage antibodies to the pan‐carcinoma marker epithelial glycoprotein‐2 for tumour targeting. Br J Cancer. 1998;78:1407–1416. 9836471 10.1038/bjc.1998.700 PMC2063226

[pro4745-bib-0036] Shiroishi M , Tsumoto K , Tanaka Y , Yokota A , Nakanishi T , Kondo H , et al. Structural consequences of mutations in interfacial Tyr residues of a protein antigen‐antibody complex: the case of HyHEL‐10‐HEL. J Biol Chem. 2007;282:6783–6791. 17166830 10.1074/jbc.M605197200

[pro4745-bib-0037] Stanfield RL , Zemla A , Wilson IA , Rupp B . Antibody elbow angles are influenced by their light chain class. J Mol Biol. 2006;357:1566–1574. 16497332 10.1016/j.jmb.2006.01.023

[pro4745-bib-0038] Tang J , Ravichandran S , Lee Y , Grubbs G , Coyle EM , Klenow L , et al. Antibody affinity maturation and plasma IgA associate with clinical outcome in hospitalized COVID‐19 patients. Nat Commun. 2021;12:1–13. 33619281 10.1038/s41467-021-21463-2 PMC7900119

[pro4745-bib-0039] Thorpe IF , Brooks CL . Molecular evolution of affinity and flexibility in the immune system. Proc Natl Acad Sci U S A. 2007;104:8821–8826. 17488816 10.1073/pnas.0610064104 PMC1885586

[pro4745-bib-0040] Yanaka S , Moriwaki Y , Tsumoto K , Sugase K . Elucidation of potential sites for antibody engineering by fluctuation editing. Sci Rep. 2017;7:9597. 28855581 10.1038/s41598-017-10246-9 PMC5577056

[pro4745-bib-0041] Yang WP , Green K , Pinz‐Sweeney S , Briones AT , Burton DR , Barbas CF . CDR walking mutagenesis for the affinity maturation of a potent human anti‐HIV‐1 antibody into the picomolar range. J Mol Biol. 1995;254:392–403. 7490758 10.1006/jmbi.1995.0626

