# Supplementary material for: Arginine cluster introduction on framework region in anti‐lysozyme antibody improved association rate constant by changing conformational diversity of CDR loops
Source: Protein Sci. 2023 Sep 1;32(9):e4745. doi: 10.1002/pro.4745 (PMC10461459; doi:10.1002/pro.4745)
Supplement: Supplementary file 1 — FIGURE S1: SPR sensorgrams for mutants with mutation in the hot‐spot residue. (a) HY33F and HY50F. (b) Additional 5‐points Arg mutation HY33F‐R5 and HY50F‐R5. (c) Alanine mutants of the hot‐spot (HY33A and HY50A) and additional 5‐points Arg mutation (HY33A‐R5 and HY50A‐R5). Running buffer injection was subtracted as the blank (gray: raw data; black: fitting data). FIGURE S2. The figure contains DSC thermogram and Tm values calculated from the analysis. R5‐mutant has a peak at lower temperature compared to wild‐type and the6 calculated Tm values were 78.3°C for wild‐type and 75.6°C for R5‐mutant, indicating the R5 mutation destabilized the Fab. FIGURE S3. Comparison of MD simulation trajectories. RMSDs of the Cα atoms between 400 ns simulations and the initial structure. Each 400 ns run was performed three times, indicated by blue, red, and green lines. Averages and standard deviations for each run are shown in the figure. Calculated in (a) total Fab molecule and (b) framework region 3 and (c) each CDR loop. (d) The distribution of the RMSD of CDR H2. FIGURE S4. Principal component analysis of each CDR loop in wild‐type and R5‐mutant. FIGURE S5. RMSF comparison with trajectory splitting. RMSF values calculated from each trajectory were overlapped. Trajectory was split into first 175 ns and last 175 ns for each simulation run. TABLE S1. Kinetic parameters for mutants of hot‐spot residue. [file PRO-32-e4745-s001.docx]

## Supporting information


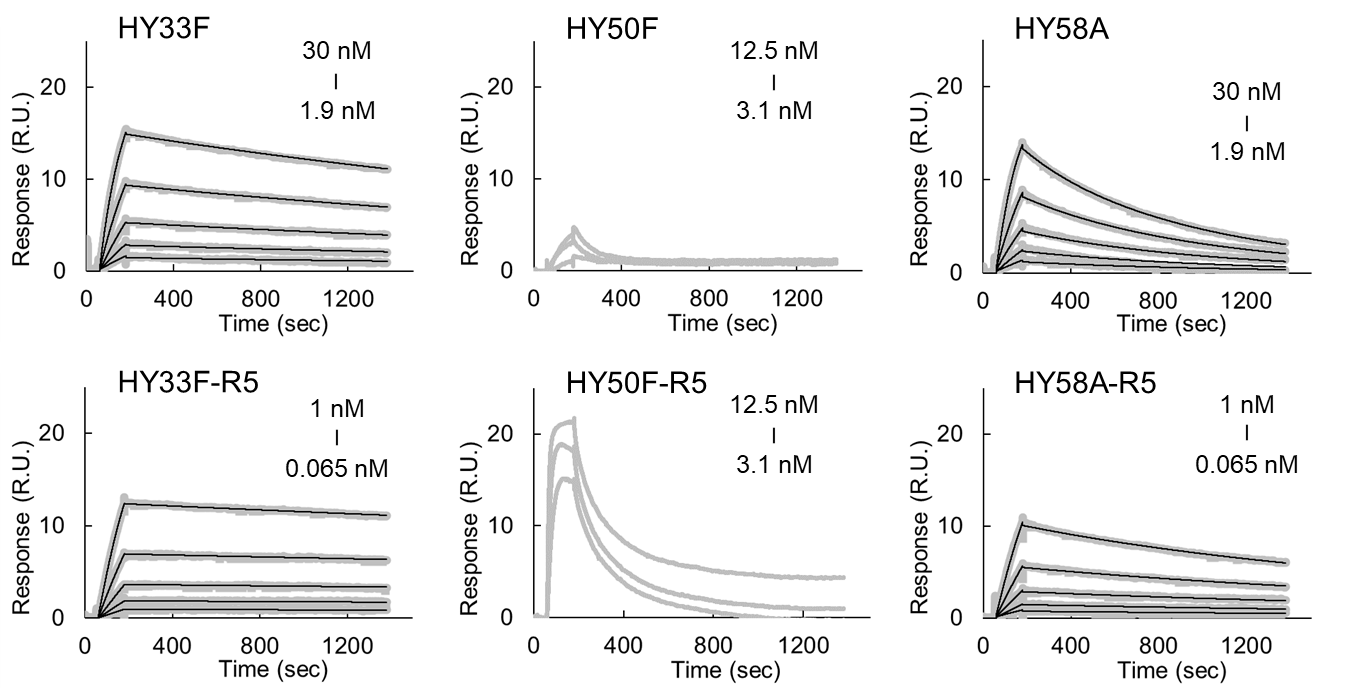
**(A)**


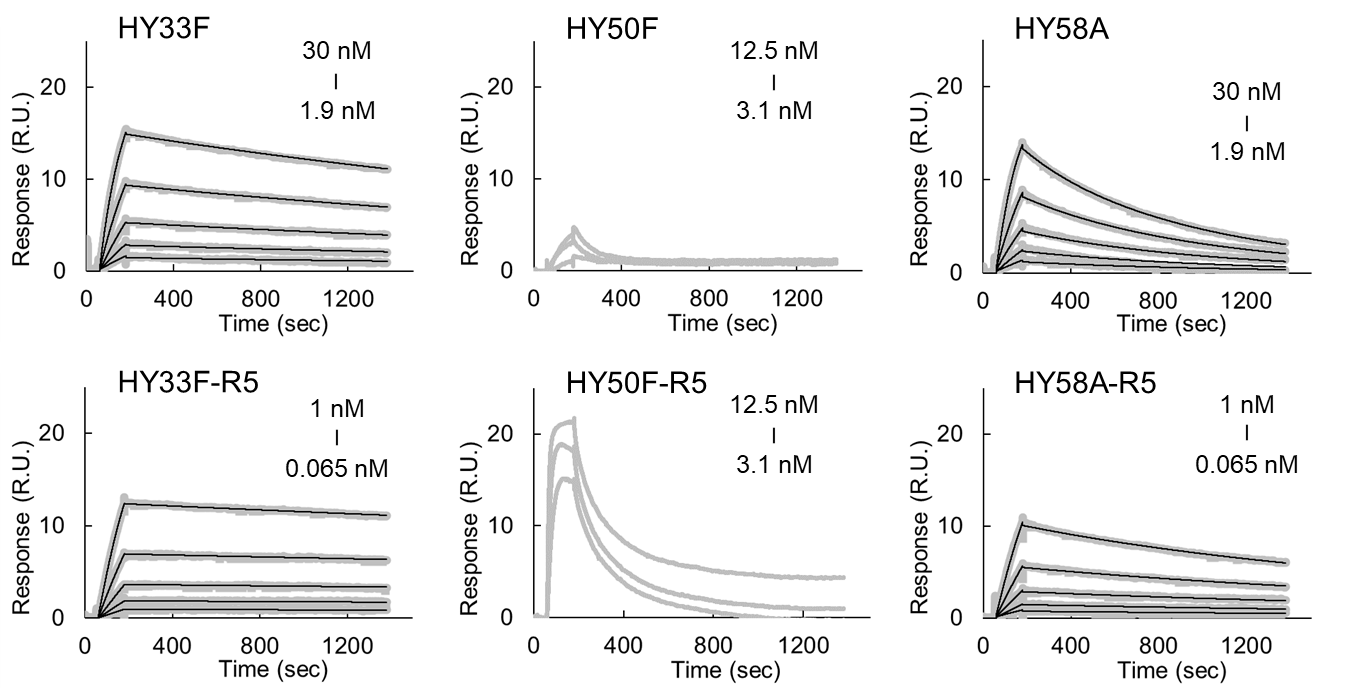
**(B)**

**
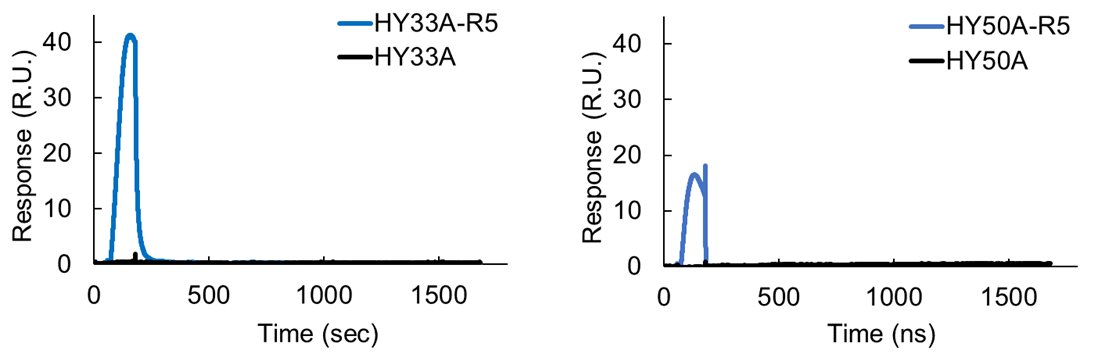
(C)**

Figure S1. SPR sensorgrams for mutants with mutation in the hot-spot residue. (A) HY33F and HY50F. (B) Additional 5-points Arg mutation HY33F-R5 and HY50F-R5. (C) Alanine mutants of the hot-spot (HY33A and HY50A) and additional 5-points Arg mutation (HY33A-R5 and HY50A-R5). Running buffer injection was subtracted as the blank. (Gray: raw data, Black: fitting Data)


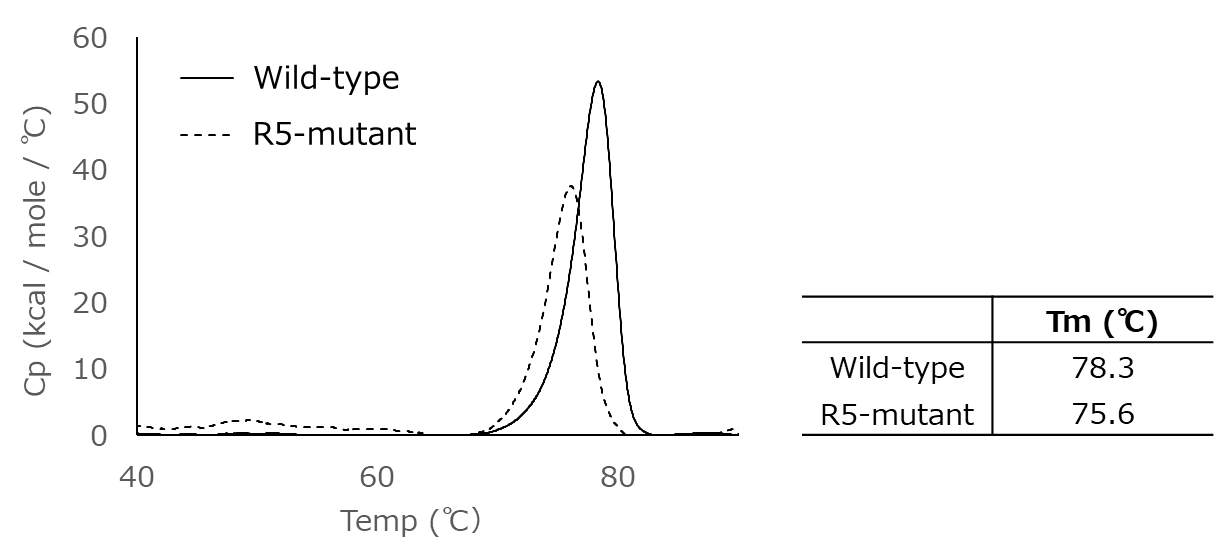


Figure S2. The figure contains DSC thermogram and Tm values calculated from the analysis. R5-mutant has a peak at lower temperature compared to wild-type and the calculated Tm values were 78.3 ºC for wild-type and 75.6 ºC for R5- mutant, indicating the R5 mutation destabilized the Fab.

(A)


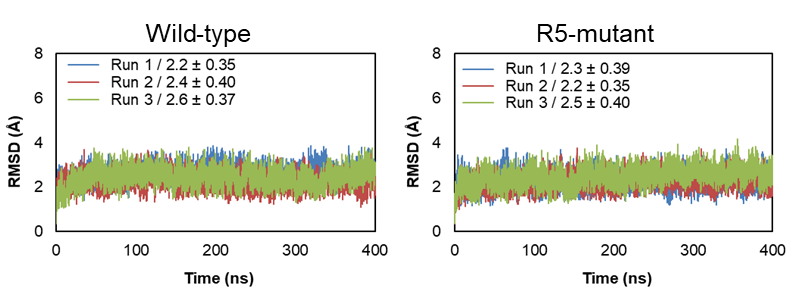


(B)


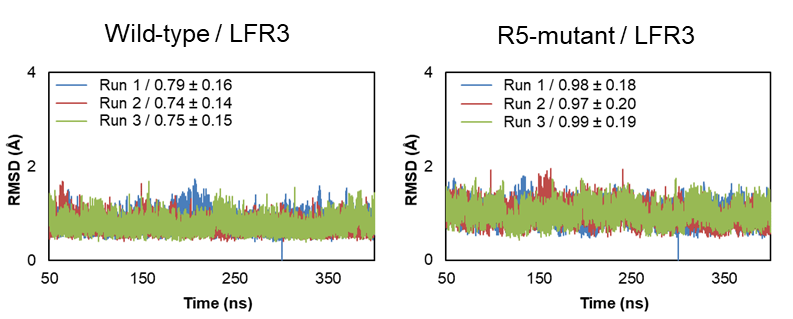


(C)


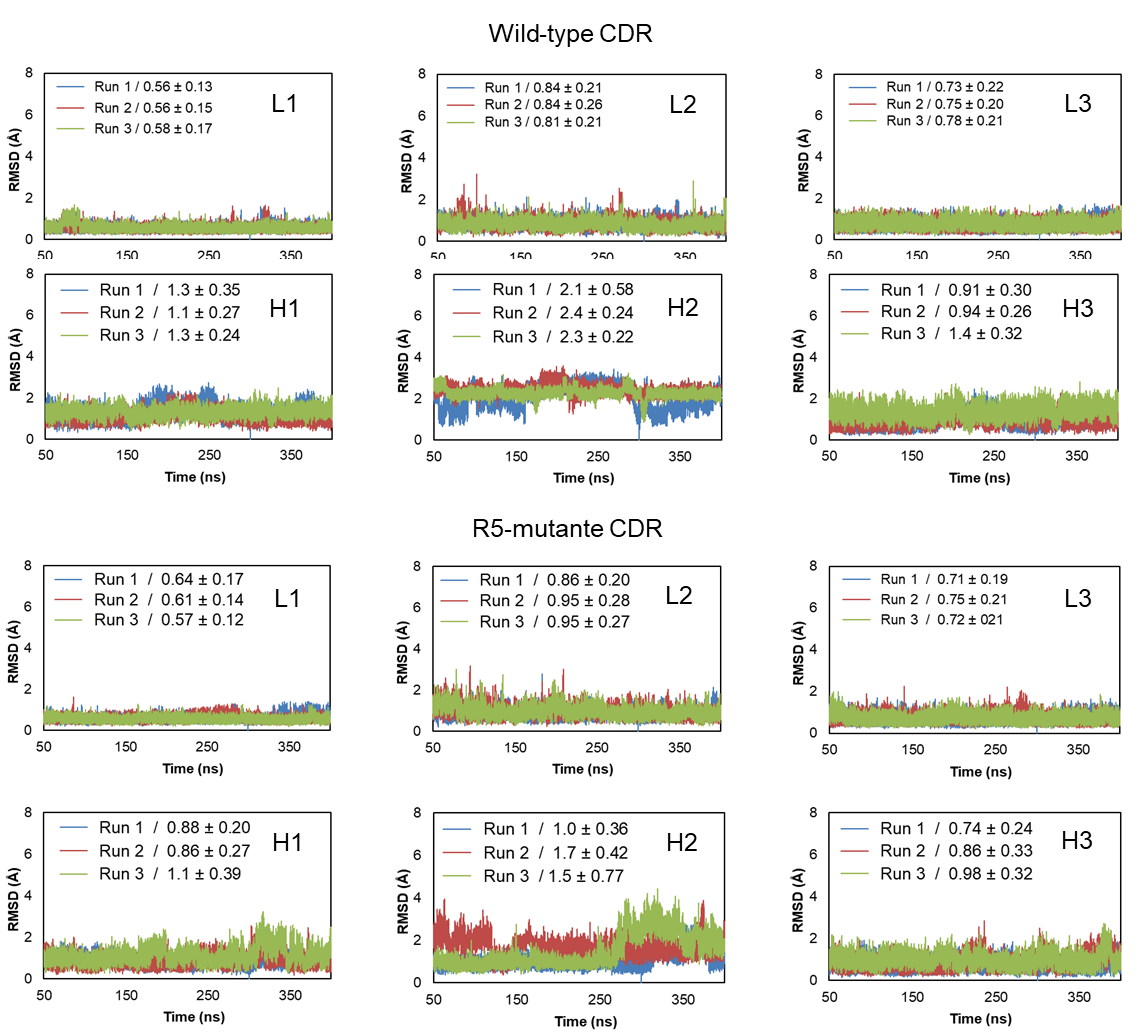


(D)


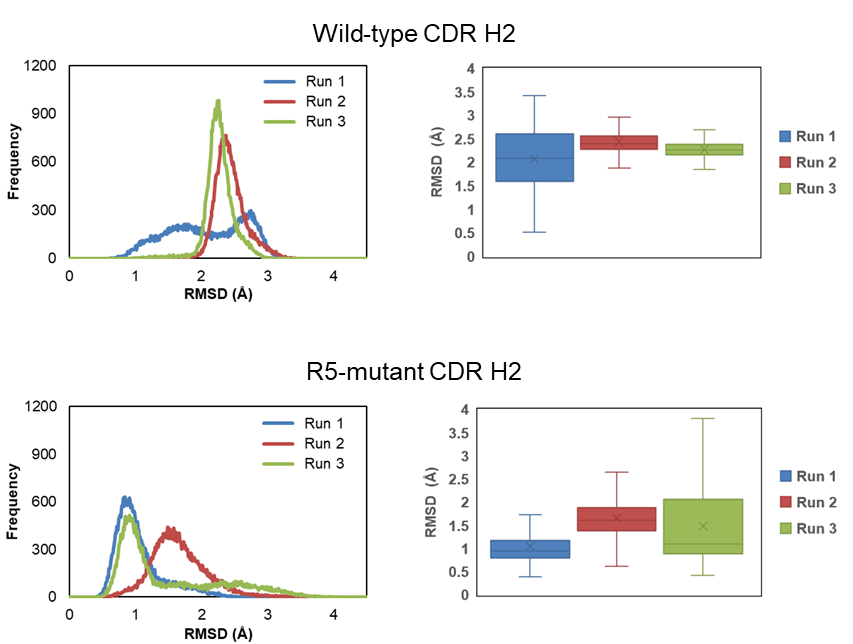


Figure S3. Comparison of MD simulation trajectories. RMSDs of the Cα atoms between 400 ns simulations and the initial structure. Each 400 ns run was performed three times, indicated by blue, red, and green lines. Averages and standard deviations for each run are shown in the figure. Calculated in (A) total Fab molecule and (B) framework region 3 and (C) each CDR loop.(D) The distribution of the RMSD of CDR H2.


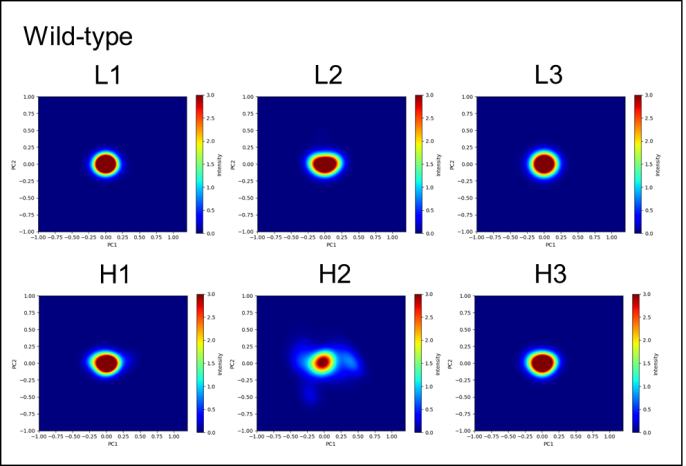

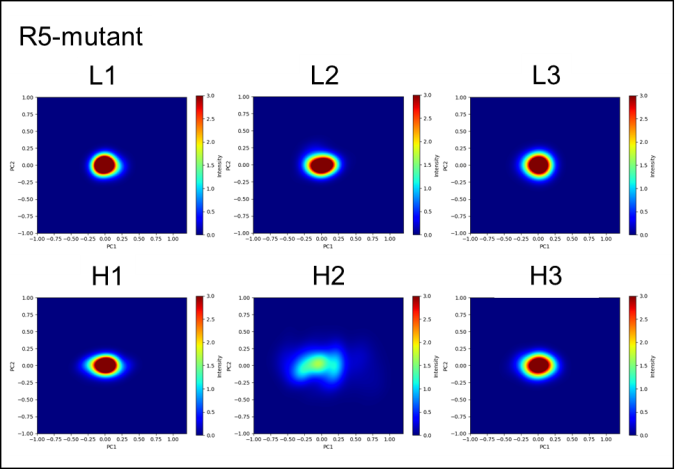


**Figure S4.** Principal component analysis of each CDR loop in Wild-type and R5- mutant.


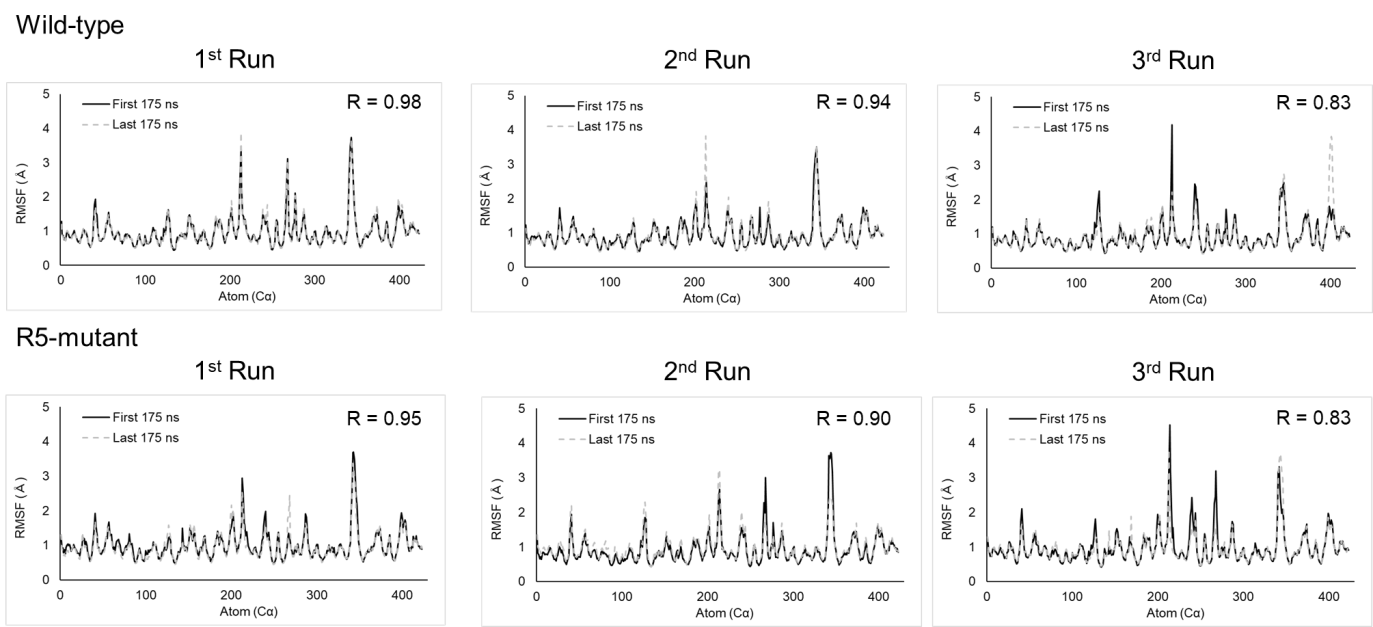


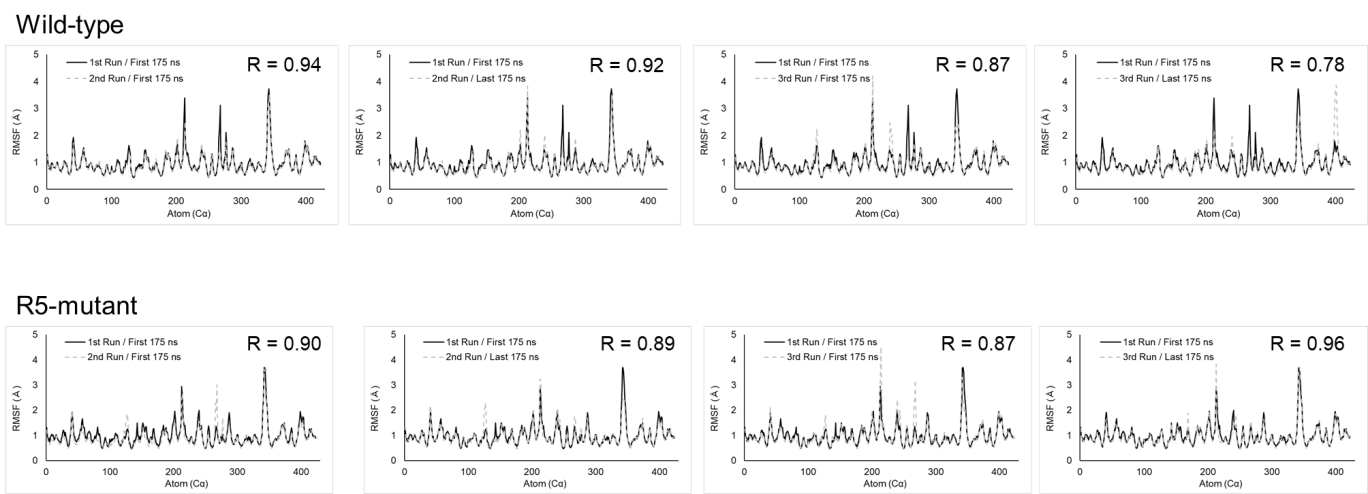


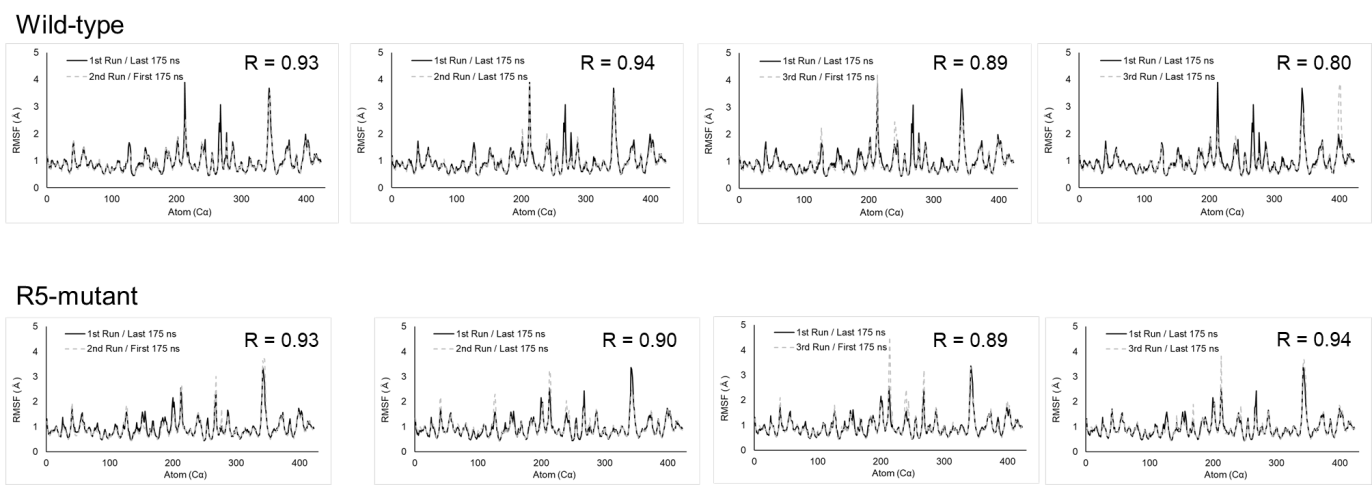


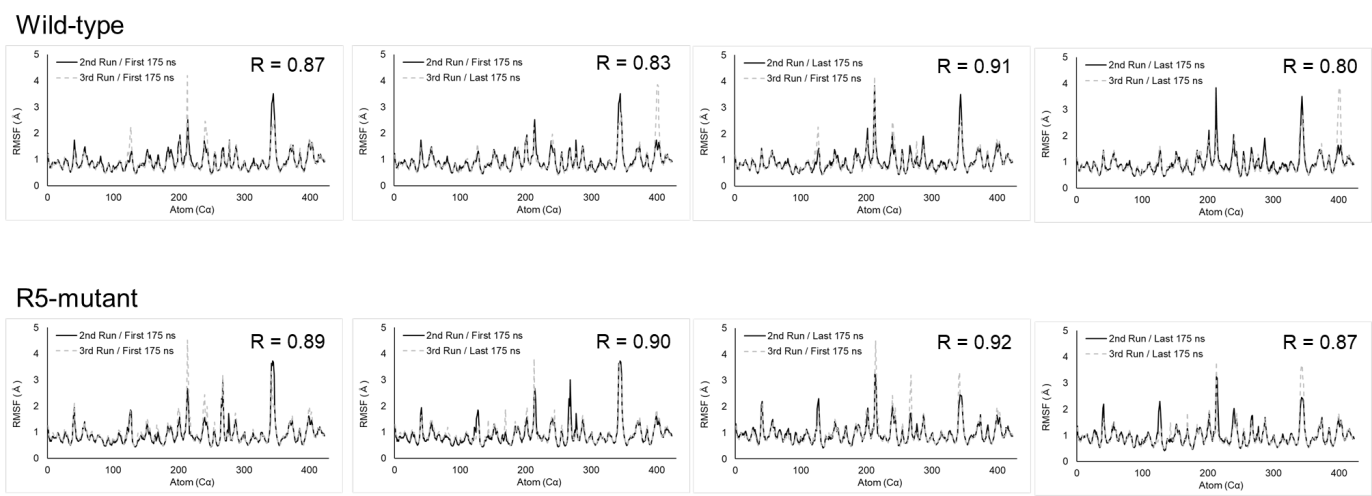


**Figure S5.** RMSF comparison with trajectory splitting. RMSF values calculated from each trajectory were overlapped. Trajectory was splitted into first 175 ns and Last 175 ns for each simulation run.

**Table S1.** Kinetic parameters for mutants of hot-spot residue

|  | ***K*_d_ (M)** | ***k*_on_ (M^−1^ s^−1^)** | ***k*_off_ (s^−1^)** |
| --- | --- | --- | --- |
| **HY33F** | 8.4 × 10^-10^ | (2.9 ±0.01) × 10^5^ | (2.4 ±0.02) × 10^-4^ |
| **HY50F** | － | － | － |
| **HY33A** | － | － | － |
| **HY50A** | － | － | － |
| **HY33F-LFR3R5** | 1.0 × 10^-11^ | (1.3 ±0.01) × 10^7^ | (1.3 ±0.01) × 10^-4^ |
| **HY50F-LFR3R5** | － | － | － |
| **HY33A-LFR3R5** | － | － | － |
| **HY50A-LFR3R5** | － | － | － |
